# Determining the contents and cell origins of apoptotic bodies by flow cytometry

**DOI:** 10.1038/s41598-017-14305-z

**Published:** 2017-10-31

**Authors:** Lanzhou Jiang, Stephanie Paone, Sarah Caruso, Georgia K. Atkin-Smith, Thanh Kha Phan, Mark D. Hulett, Ivan K. H. Poon

**Affiliations:** Department of Biochemistry and Genetics, La Trobe Institute for Molecular Science, La Trobe University, Victoria, 3086 Australia

## Abstract

Over 200 billion cells undergo apoptosis every day in the human body in order to maintain tissue homeostasis. Increased apoptosis can also occur under pathological conditions including infection and autoimmune disease. During apoptosis, cells can fragment into subcellular membrane-bound vesicles known as apoptotic bodies (ApoBDs). We recently developed a flow cytometry-based method to accurately differentiate ApoBDs from other particles (e.g. cells and debris). In the present study, we aim to further characterize subsets of ApoBDs based on intracellular contents and cell type-specific surface markers. Utilizing a flow cytometry-based approach, we demonstrated that intracellular contents including nuclear materials and mitochondria are distributed to some, but not all ApoBDs. Interestingly, the mechanism of ApoBD formation could affect the distribution of intracellular contents into ApoBDs. Furthermore, we also showed that ApoBDs share the same surface markers as their cell of origin, which can be used to distinguish cell type-specific ApoBDs from a mixed culture. These studies demonstrate that ApoBDs are not homogeneous and can be divided into specific subclasses based on intracellular contents and cell surface markers. The described flow cytometry-based method to study ApoBDs could be used in future studies to better understand the function of ApoBDs.

## Introduction

Apoptosis is a major form of cell death under normal physiological settings^1,2^. At later stages of apoptosis, cells can disassemble and generate subcellular (generally 1–5 μm in diameter) membrane-bound extracellular vesicles termed apoptotic bodies (ApoBDs)^3,4^. Like exosomes and microvesicles, ApoBDs are classified as a type of extracellular vesicle (EV)^5^, which can be generated from many (but not all) cell types, including T cells, monocytes, fibroblasts, endothelial cells and epithelial cells^6–8^. The formation of ApoBDs has been proposed to play an important role in the clearance of apoptotic cells by phagocytes^9^. It has also been shown that ApoBDs can carry DNA, microRNAs, proteins and lipids to mediate communication between cells^10–12^.

The fragmentation of an apoptotic cell is not a stochastic process as previously assumed. It is becoming apparent that the generation of ApoBDs is controlled by several distinct morphological steps, a process known as apoptotic cell disassembly^4,13–16^. The disassembly process can be divided into three key steps: (Step 1) formation of plasma membrane blebs on the cell surface, (Step 2) generation of apoptotic membrane protrusions (e.g. microtubule spikes, apoptopodia and beaded apoptopodia), and (Step 3) fragmentation which leads to the formation of individual ApoBDs^4,14,15^. These steps are regulated by distinct molecular factors, such as the Rho-associated protein kinase ROCK1^17,18^ and the plasma membrane channel pannexin 1 (PANX1)^15^.

Conventionally, the study of ApoBDs relies heavily on histological and confocal microscopy analyses of tissue samples and cells undergoing cell death *in vitro*, respectively. Recently, we established a flow cytometry-based method to measure ApoBD formation in cell culture samples based on particle size and granularity, as well as annexin A5 (A5) binding (a measure of phosphatidylserine exposure) and nucleic acid dye TO-PRO-3 uptake (dependent on PANX1 opening and membrane permeability, stains early apoptotic cells and necrotic cells differentially)^19^. Developing methodologies to accurately identify ApoBDs in a complex sample is critical for elucidating the mechanisms and functions of apoptotic cell disassembly. In this study, we investigated whether ApoBDs can be further divided into different subsets based on other characteristics such as intracellular contents and cell surface markers. First, we examined the distribution of DNA, RNA and mitochondria in ApoBDs and found that certain intracellular contents were localized to some, but not all ApoBDs. The distribution of intracellular contents into ApoBDs is associated with the mechanism of apoptotic cell disassembly. Next, we investigated if ApoBDs bear similarity to their cell of origin based on cell type-specific surface marker expression. We found that ApoBDs express surface markers from their cell of origin which can be used to identify ApoBDs of a particular cell type in a mixed culture.

## Materials and Methods

### Reagents

Trovafloxacin (Sigma-Aldrich, USA, PZ0015), GSK 269962 (Tocris bioscience, UK, 4009), Hoechst 33342 (Sigma-Aldrich, St Louis, MO). SYTO RNASelect green Fluorescent Cell Stain (S32703), MitoTracker Green (M7514), TO-PRO-3 (T3605) were purchased from Thermo Fisher Scientific. CD45-PeCy7 (557748; clone: HI30), CD3-APC (555335; clone: UCHT1), IgG1κ-PeCy7 (557872; clone: MOPC-21), A5-FITC (556419), A5-PE (556421), A5-APC (550474), A5-V450 (560506) and 10 × A5 binding buffer (556454) were purchased from BD Biosciences, CA. CD146-VioBlue (130-099-678; clone: 541-10B2), CD31-VioBlue (130-106-503; clone: AC128), CD45-FITC (130-080-202; clone: 5B1), CD14-FITC (130-080-701; clone: TÜK4), CD11b-FITC (130-081-201; clone: M1/70.15.11.5), IgG1-VioBlue (130-099-756), IgG2b-FITC (130-103-088), IgG2a-FITC (130-091-837), IgG1-FITC (130-098-847), IgG1-APC (130-098-846) were purchased from MAC Miltenyi Biotech, DE.

### Mammalian cell culture

Human THP-1 monocytic, Jurkat T cells and HL-60 promyelocytic leukemia cells were obtained from ATCC and cultured in complete RPMI media. Culture media was prepared using RPMI 1640 medium (Life Technologies, 22400-089), supplemented with 10% (vol/vol) fetal bovine serum (FBS, Gibco, USA, 10099-141), penicillin (50 U/mL) and streptomycin (50 mg/mL) mixture (Life Technologies, 15140122) and 0.2% (vol/vol) MycoZap (Lonza, Switzerland, LT07-818). For HL-60 cells, 2 mM L-glutamine (100 U/mL) was also added to the culture media. Human Umbilical Vein Endothelial Cells (HUVEC) were obtained from Lonza and cultured in Clonetics^TM^ EGM^TM^-2 BulletKit^TM^ composed of EBM^TM^ basal medium, 0.2% (vol/vol) MycoZap and the following growth supplements: human epidermal growth factor, vascular endothelial growth factor, R3- insulin-like growth factor-1, ascorbic acid, hydrocortisone, human fibroblast growth factor-beta, heparin, FBS and gentamycin/Amphotericin-B. All mammalian cell lines were cultured at 37 °C in a humidified atmosphere with 5% CO_2_ at all times.

### Induction of apoptosis

HUVEC were seeded in 100 mm tissue culture dishes and allowed to adhere overnight. THP-1, Jurkat and HL-60 cells were incubated in 6-well culture plates or in FACS tubes while inducing apoptosis. Apoptosis was induced by ultraviolet (UV) irradiation with a Stratagene UV Stratalinker 1800 (Agilent Technologies, CA) at 150 mJ/cm^2^ or by anti-Fas treatment (62.5 ng/ml). THP-1 cells, Jurkat cells and HUVEC were incubated at 37 °C in a humidified atmosphere with 5% CO_2_ for 2 to 6 h. Primary thymocytes (collected from 4- to 5-week-old C57BL/6 mice, female) were treated with 50 μM dexamethasone for 4 h. During apoptosis induction, all cell types were incubated in 1% BSA/RPMI media.

### Monitoring the distribution of intracellular contents by flow cytometry

Samples were stained with a combination of Hoechst 33342/A5-FITC/TO-PRO-3 to examine DNA distribution, MitoTracker Green/A5-V450/TO-PRO-3 to examine mitochondria distribution, Hoechst 33342/MitoTracker Green/A5-APC to examine DNA and mitochondria distribution at the same time, or Hoechst 33342/SYTO RNAselect green/A5-APC to examine DNA and RNA distribution at the same time. Cells were stained with Hoechst 33342 (5 μg/ml), MitoTracker Green (100 nM) and/or SYTO RNAselect green (500 nM) prior to induction of apoptosis. Cells were stained with A5 and/or TO-PRO-3 in 1 × A5 binding buffer for 10 min at room temperature and immediately placed on ice before analysis on a FACSCanto flow cytometer and FACSDiva 6.1.1 software (BD Bioscience). Unstained samples were used as a control. All data were analyzed using FlowJo 8.8.6 and 8.8.10 software (Tree Star).

### Monitoring the localization of intracellular contents by confocal microscopy

THP-1 and Jurkat cells were stained with MitoTracker Green (100 nM) and Hoechst 33342 (5 μg/ml) before induction of apoptosis. Jurkat and HL-60 cells were stained with Hoechst 33342 (5 μg/ml) and SYTO RNAselect green (500 nM). Samples were incubated in Lab-TekII 4-well chamber slides (Nunc) and confocal microscopy was performed at 37 °C in a humidified atmosphere with 5% CO_2_ using Zeiss LSM780 Laser Scanning Confocal Microscope (Carl Zeiss SAS, Jena, Germany) to monitor the progression of ApoBD formation within 4 hours.

### Imaging A5 stained apoptotic bodies and apoptotic cells by confocal microscopy

Jurkat cells were stained with SYTO RNAselect Green, MitoTracker Green and/or Hoechst 33342 before induction of apoptosis. After 4  h of incubation, samples were stained with A5-FITC/A5-V450 or A5-APC in 1 × A5 binding buffer for 10 min at room temperature in dark. The samples are prepared the same way as for flow cytometry. Samples were then diluted in RPMI/1%BSA and transferred to Poly-L-lysine coated Lab-TekII 8-well chamber slide system. Samples were allowed to settle onto the chamber slide for 10 min prior to imaging. Images were taken using a Zeiss LSM780 Laser Scanning Confocal Microscope.

### Monitoring surface markers by flow cytometry and confocal microscopy

To establish optimal surface markers for various cell types, viable THP-1 and Jurkat cell samples were collected from suspended culture. Viable, adherent HUVEC were collected after 0.5% EDTA treatment. Cells were pelleted at 1000 *g*, then resuspended in serum free RPMI containing one of CD146-VioBlue (1:50), CD31-VioBlue (1:50), CD14-FITC (1:100), CD45-FITC (1:100), CD3-APC (1:50), CD11b-FITC (1:50). Isotype controls included IgG1-VioBlue (1:50), IgG2a-FITC (1:100), IgG1-FITC (1:100), IgG1-APC (1:50), IgG2b-FITC (1:50) and incubated for 20 min on ice. Samples were pelleted and resuspended in serum free RPMI for analysis by flow cytometry. Although Fc-block was not used and did not have a major impact on unspecific binding of antibodies used in this study (data not shown), a Fc-block step is recommended for antibodies with high unspecific binding to the cell type of interest.

To investigate surface marker changes during apoptosis, viable THP-1 cells, Jurkat cells and HUVEC were collected as outlined above as a control. UV-treated whole apoptotic samples of THP-1 and Jurkat cells were collected, while only the supernatant of UV-treated HUVEC containing apoptotic cells and ApoBDs was collected. Samples were pelleted at 1000 *g*, resuspended in 1 × A5 binding buffer containing A5-PE (1:1000) and incubated for 10 min at room temperature. Samples were pelleted at 1000 *g*, resuspended in an antibody cocktail containing CD146-VioBlue (1:50), CD45-PECy7 (1:100), CD3-APC (1:100), CD11b-FITC (1:50) in 1 × A5 binding buffer and incubated for 20 min on ice. Isotype controls included IgG1-VioBlue (1:50), IgG1κ-PeCy7 (1:100), IgG1-APC (1:100) and IgG2b-FITC (1:50). Samples were pelleted at 1000 *g* and resuspended in 1 × A5 binding buffer for analysis by flow cytometry. Compensation was performed using single stain controls.

To establish a mixed culture, apoptotic THP-1 monocytes and Jurkat T cells were added to the supernatant of apoptotic HUVEC. Mixed culture samples were pelleted at 1000 *g*, resuspended in 1 × A5 binding buffer containing A5-PE (1:1000) and incubated for 10 min at room temperature. Samples were pelleted at 1000 *g*, resuspended in an antibody cocktail containing CD45-PeCy7 (1:50), CD146-VioBlue (1:50), CD3-APC (1:100), CD11b-FITC (1:50) in 1 × A5 binding buffer and incubated for 20 min on ice. Samples were pelleted at 1000 *g* and resuspended in 1 × A5 binding buffer for analysis by flow cytometry. Compensation was performed using single stain controls.

To monitor cell surface markers on ApoBDs by confocal microscopy, ApoBDs were enriched by a previously established centrifugation method^8^. Briefly, apoptotic supernatant was centrifuged at 300 *g* for 10 min to pellet cells, and resulting supernatant was centrifuged at 3000 *g* for 20 min to pellet ApoBDs. HUVEC, THP-1 and Jurkat ApoBDs were resuspended in serum free RPMI containing CD146-VioBlue (1:50), CD45-FITC (1:50) or CD3-APC (1:50) respectively and incubated for 20 min on ice. ApoBDs were pelleted at 1000 *g* for 5 min, then resuspended in serum free RPMI and added to Lab-TekII 4-well chamber slides for microscopy analysis. Images were acquired using the Zeiss LSM780 Laser Scanning Confocal Microscope.

### Statistical analyses

The data are presented as means ± standard error of the mean (s.e.m.). All data presented are representative of at least three independent experiments. Statistical analyses were performed using Student’s two-tailed t-test. A *P*-value of less than 0.05 was considered statistically significant. *P < 0.05, **P < 0.01, ***P < 0.001.

## Results

### Different subsets of ApoBDs can be identified based on intracellular contents by flow cytometry

**Figure 1:**
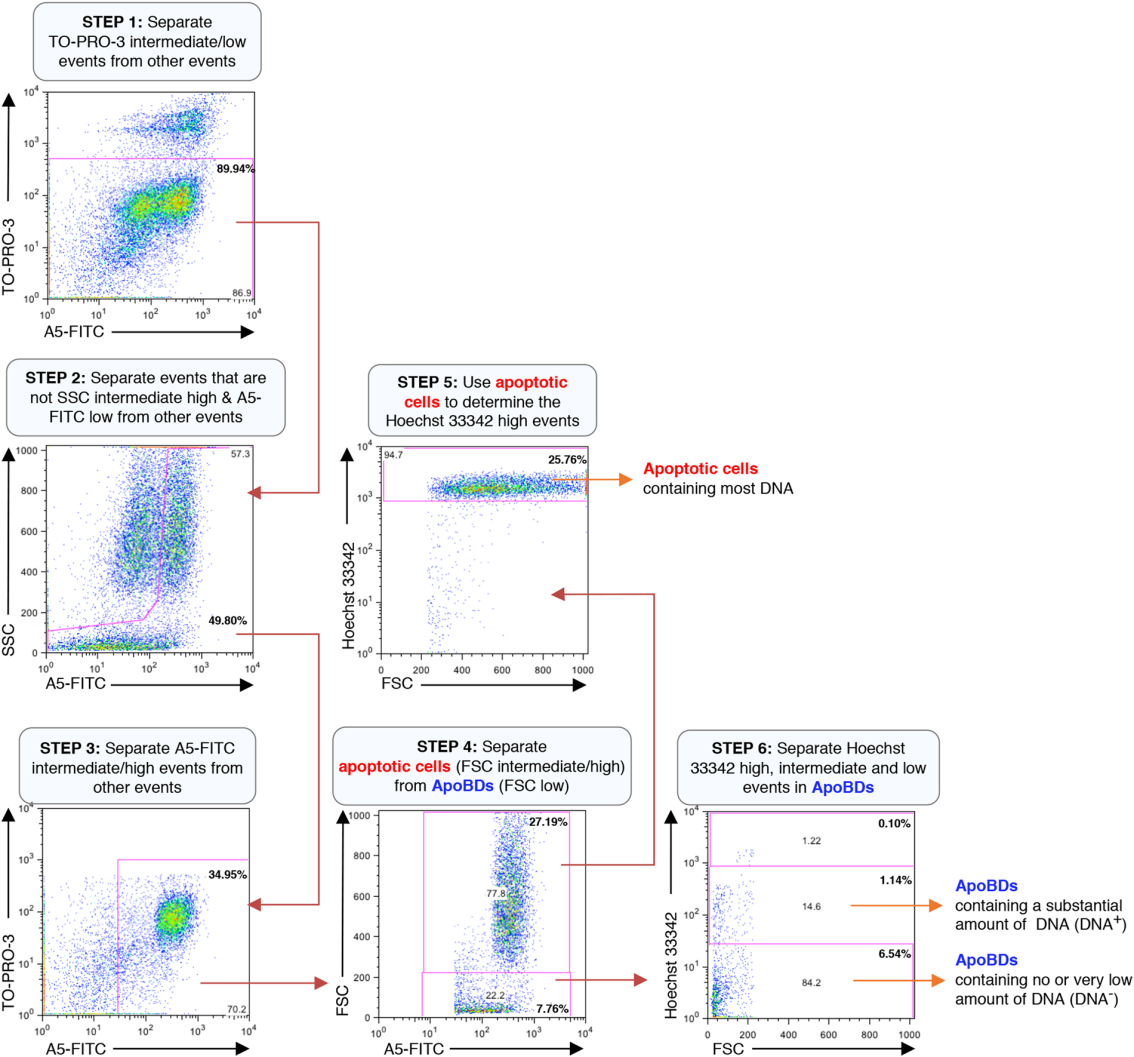
Electronic gating strategy for analysis of DNA content in ApoBDs generated from apoptotic Jurkat T cells. Flow cytometry analysis showing the six-stage electronic gating strategy used to identify ApoBDs with different amount of DNA from a sample containing a mixture of viable cells, apoptotic cells, necrotic cells, cell debris and ApoBDs. The final percentages of ApoBDs containing different amount of DNA relative to all the cells/particles in the sample are included on the flow cytometry plots (bold). The gating of Hoechst 33342 low events is determined based on unstained sample. Data are representative of at least three independent experiments.

**Figure 2:**
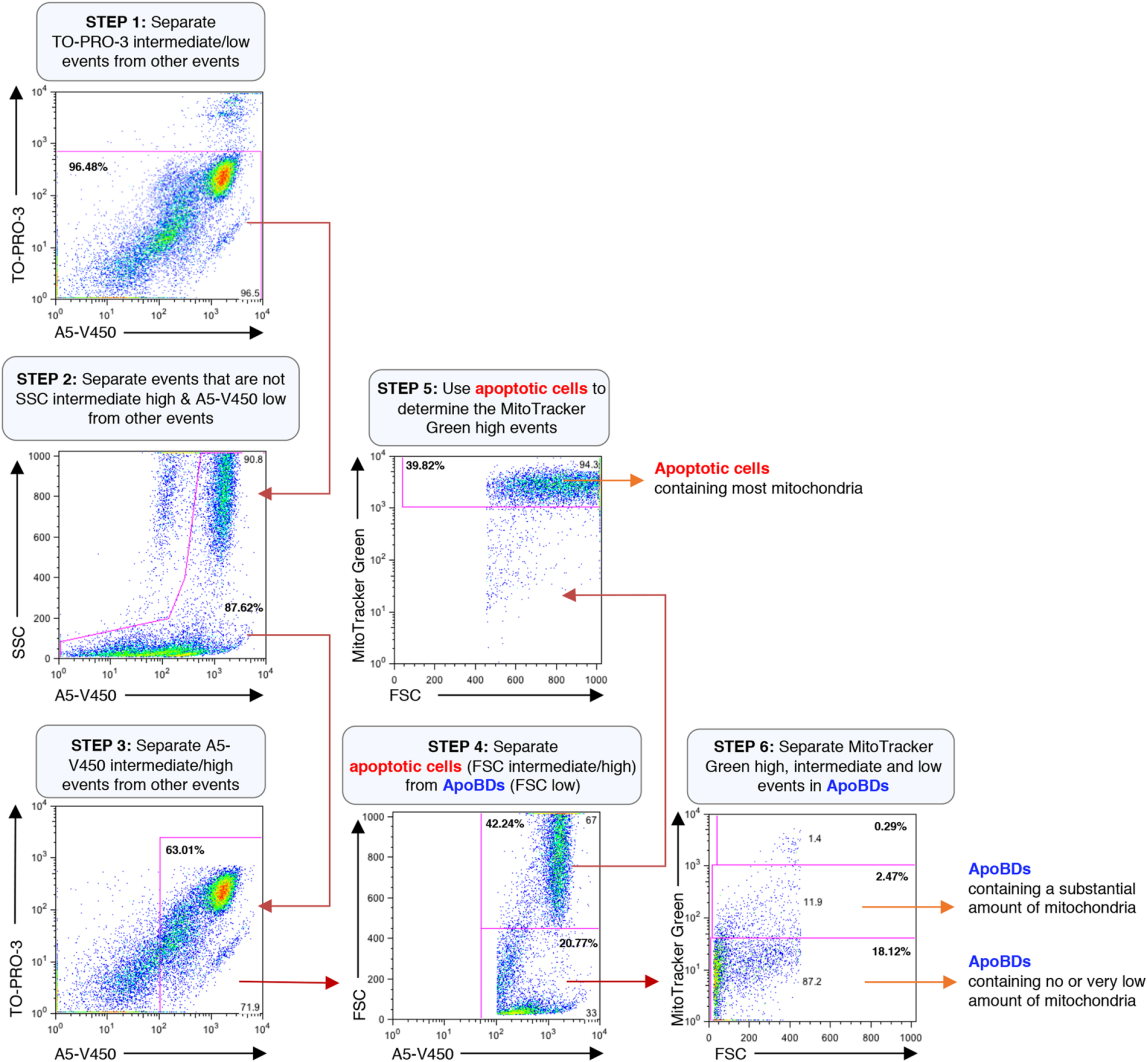
Electronic gating strategy for analysis of mitochondrial content in ApoBDs generated from apoptotic Jurkat T cells. Flow cytometry analysis showing the six-stage electronic gating strategy used to identify ApoBDs with different amount of mitochondria from a sample containing a mixture of viable cells, apoptotic cells, necrotic cells, cell debris and ApoBDs. The final percentages of ApoBDs containing different amount of mitochondria relative to all the cells/particles in the sample are included on the flow cytometry plots (bold). The gating of MitoTracker Green low events is determined based on unstained sample. Data are representative of at least three independent experiments.

During the progression of apoptosis, cells can dismantle by packaging organelles such as the Golgi apparatus, ER and condensed chromatin into ApoBDs^20,21^. We asked whether the distribution of different intracellular contents in ApoBDs can be monitored by flow cytometry. First, we monitored the distribution of nuclear contents by staining human Jurkat T cells with the cell-permeable DNA binding dye Hoechst 33342 prior to induction of apoptosis. Next, cells were induced to undergo apoptosis by UV or anti-Fas treatment. After 4 h of incubation to allow for cells to undergo apoptosis and cell disassembly^14^, cells were stained with the nucleic acid dye TO-PRO-3 (stains early apoptotic cells and necrotic cells differentially) and A5-FITC (measures phosphatidylserine exposure), and flow cytometry analysis was performed to differentiate ApoBDs by the electronic gating strategy as shown in Fig. 1. Induction of apoptosis was also confirmed under these conditions (Supplementary Fig. 1a). Utilizing this approach, ApoBDs can be further separated into two distinct subsets based on Hoechst 33342 staining, which measures the amount of DNA contained in ApoBDs. The two subsets of ApoBDs are those that contain a substantial amount of DNA, and those that contain no or very low amounts of DNA (Fig. 1). The presence of these two subsets of ApoBDs was confirmed by confocal microscopy (Supplementary Fig. 2a). Although another small population of ApoBDs seems to contain a comparable amount of DNA as whole apoptotic cells, these particles exhibited a relatively higher FSC (i.e. particle size) and were excluded from the analysis as they may represent apoptotic cells rather than ApoBDs (Fig. 1). Similarly, the distribution of mitochondria into ApoBDs can also be monitored by staining Jurkat T cells with MitoTracker Green prior to induction of apoptosis. For this approach, a combination of TO-PRO-3 and A5-V450 were used to identify ApoBDs in the sample, and mitochondrial contents in ApoBDs determined by the electronic gating strategy as shown in Fig. 2. ApoBDs can be separated into two subsets based on their MitoTracker Green staining. The two subsets of ApoBDs are those that contain a substantial amount of mitochondria and those that contain no or very low amounts of mitochondria (Fig. 2). The presence of these two subsets of ApoBDs can be confirmed by confocal microscopy (Supplementary Fig. 2b). These data support the concept that ApoBDs can be separated into different subsets based on their intracellular contents, and the distribution of intracellular contents like DNA and mitochondria into ApoBDs can be efficiently monitored by the described flow cytometry-based approach.

### Distribution of intracellular contents into ApoBDs is associated with the mechanism of apoptotic cell disassembly

**Figure 3:**
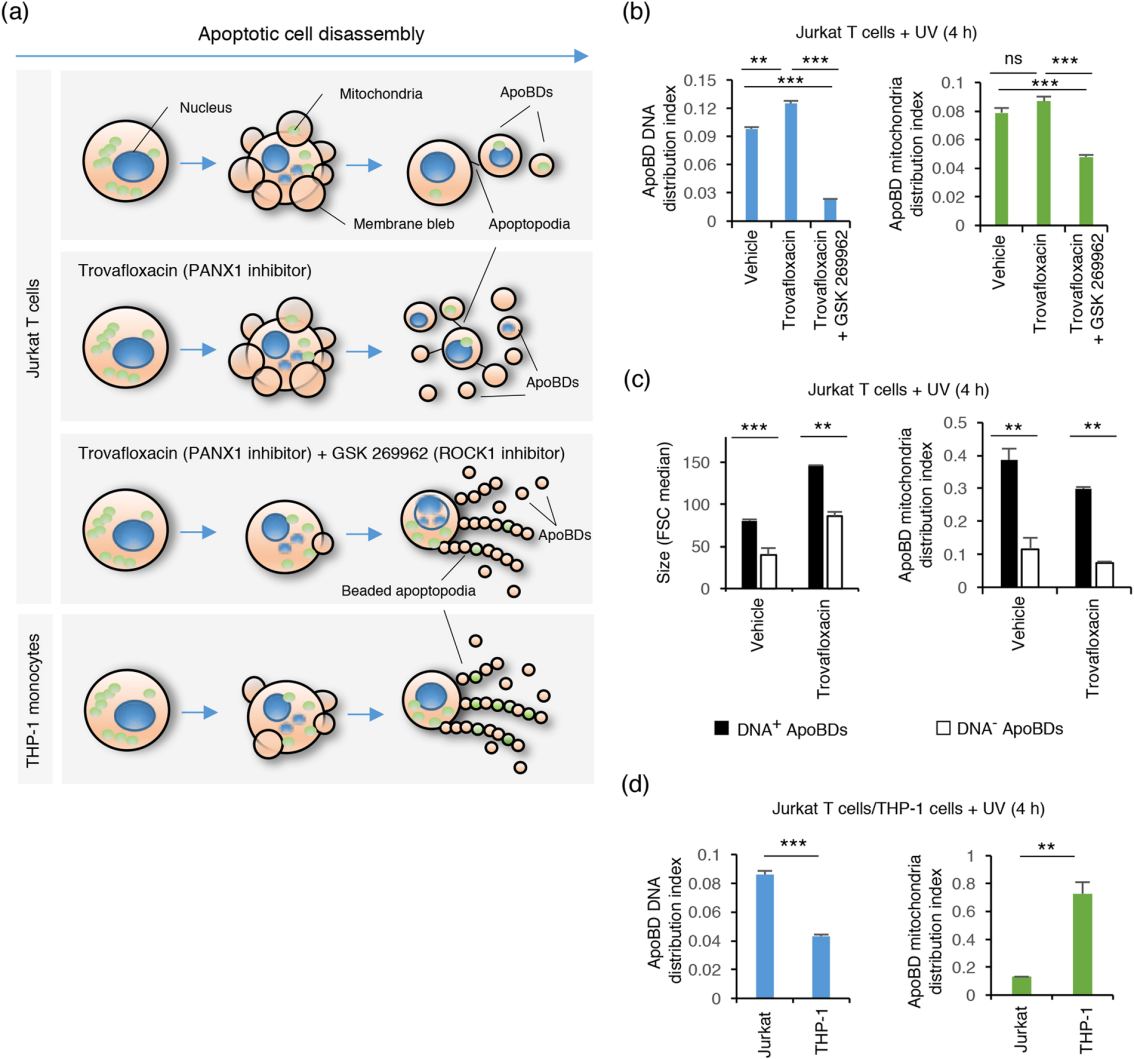
Distribution of intracellular contents into ApoBDs is altered by the mechanism of apoptotic cell disassembly. (**a**) Top, schematic of Jurkat T cells undergoing apoptotic cell disassembly and the distribution of intracellular contents (DNA and mitochondria) when treated with trovafloxacin (PANX1 inhibitor) or a combination of trovafloxacin and GSK 269962 (ROCK1 inhibitor) during apoptosis. Bottom, schematic of THP-1 monocytes undergoing apoptotic cell disassembly and the distribution of intracellular contents during this process. (**b**) Jurkat T cells treated with trovafloxacin (20 μM) alone or in combination with GSK 269962 (1 μM) to modulate the mechanism of ApoBD formation. The distribution of DNA and mitochondria into ApoBDs quantified based on ApoBD DNA distribution index and ApoBD mitochondria distribution index, respectively (n = 3). ApoBD DNA distribution index = DNA^+^ ApoBDs/DNA^−^ ApoBDs; ApoBD mitochondria distribution index = mitochondria^+^ ApoBDs/mitochondria^−^ ApoBDs. (**c**) Jurkat T cells were treated with trovafloxacin (20 μM) to modulate the mechanism of ApoBD formation, and the size of ApoBDs (based on forward scatter, FSC) and distribution of mitochondria into ApoBDs (based on ApoBD mitochondria distribution index) were determined for ApoBDs that contain a substantial amount (DNA^+^), or no or very low amount of DNA (DNA^−^) (n = 3) (**d**) Comparison of the distribution of DNA and mitochondria into ApoBDs generated from apoptotic Jurkat T cells and THP-1 monocytes (n = 3). (**b–d**) Jurkat T cells and THP-1 monocytes were treated with UV to induce apoptosis. Data are representative of at least three independent experiments. *P < 0.05, **P < 0.01, ***P < 0.001, unpaired Student’s two-tailed t-test.

Recent studies have shown that the formation of ApoBDs is a highly regulated process controlled by different morphologies and molecular factors^4,13–15^ (Fig. 3a). In this study, we asked whether the distribution of intracellular contents such as DNA and mitochondria (monitored as per above) into ApoBDs could be influenced by the mechanism of apoptotic cell disassembly. First, we manipulated the mechanism of ApoBD formation by targeting key regulators such as the kinase ROCK1 and the membrane channel PANX1^15,17^. For apoptotic T cells, caspase-activated ROCK1 is a key positive regulator of cell disassembly by driving membrane blebbing^17,18^, and caspase-activated PANX1 is a negative regulator of apoptopodia formation, by promoting the separation of membrane blebs to generate distinct ApoBDs^15^. In our recent studies using Jurkat T cells as a model, we demonstrated that PANX1 blockade could enhance ApoBD formation by promoting the separation of membrane blebs through apoptopodia, whereas blockade of both ROCK1 and PANX1 could promote ApoBD formation via the generation of a ‘beads-on-a-string’ membrane protrusion (known as beaded apoptopodia) (Fig. 3a)^13^. Taking advantage of this system, we were able to manipulate the mechanism of apoptotic cell disassembly by treating Jurkat T cells with PANX1 inhibitor (trovafloxacin) or a combination of ROCK1 and PANX1 inhibitors (GSK 269962 and trovafloxacin, respectively) during apoptosis (Fig. 3a).

Initially, changes in DNA distribution into ApoBDs were quantified based on ApoBD DNA distribution index (i.e. the number of ApoBDs containing a substantial amount of DNA divided by the number of ApoBDs containing no or very low amounts of DNA). When Jurkat T cells were treated with trovafloxacin to promote apoptopodia-mediated ApoBD formation during apoptosis, the ApoBD DNA distribution index increased significantly (Fig. 3b), indicating an increase in ApoBDs containing a substantial amount of DNA. In contrast, when Jurkat T cells were treated trovafloxacin and GSK 269962 to promote beaded apoptopodia-mediated ApoBD formation, a marked reduction in ApoBD DNA distribution index was observed (Fig. 3b), indicating an increase in ApoBDs containing no or very low amounts of DNA. These observations were also confirmed by confocal microscopy (Supplementary Fig. 1b). It should be noted that similar results were observed when Jurkat T cells were induced to undergo apoptosis by either UV or anti-Fas treatments (Fig. 3b, Supplementary Fig. 1e) and primary mouse thymocytes induced to undergo apoptosis by dexamethasone (Supplementary Fig. 1f), indicating that these changes in DNA distribution in ApoBDs is independent of apoptotic stimulus. Interestingly, the distribution of mitochondria into ApoBDs showed a different trend compared to DNA when the disassembly of apoptotic cells was modulated by pharmacological compounds. Trovafloxacin treatment had no significant effects on the number of ApoBDs containing a substantial amount of mitochondria, whereas a combination of trovafloxacin and GSK 269962 treatment reduced ApoBD mitochondria distribution index, indicating an increase in ApoBDs containing no or very low amounts of mitochondria (Fig. 3b). Collectively, these data suggest that the mechanism used by the cell to undergo apoptotic cell disassembly could alter the distribution of intracellular contents into ApoBDs, and not all intracellular contents are affected to the same extent. It should be noted that these results correlated well with confocal microscopy images of Jurkat T cells stained with both Hoechst 33342 and MitoTracker Green undergoing apoptotic cell disassembly under different conditions as described above (Supplementary Fig. 1b). In addition, it is interesting to note that ApoBDs containing a substantial amount of DNA are often higher in FSC (indicative of size by flow cytometry analysis) and vesicle diameter (by confocal microscopy analysis) compared with ApoBDs containing no or very low amounts of DNA (Fig. 3c and Supplementary Fig. 3a). Moreover, to monitor the co-distribution of both DNA and mitochondria into ApoBDs, Jurkat T cells were stained with a combination of Hoechst 33342 and MitoTracker Green before apoptosis induction, and stained with A5-APC prior to flow cytometry analysis. Interestingly, ApoBDs containing a substantial amount of DNA also harbor more mitochondrial content compared to ApoBDs containing no or very low amounts of DNA (Fig. 3c).

In our recent studies, we have demonstrated that different cell types can generate ApoBDs via different mechanisms. In particular, Jurkat T cells and THP-1 monocytic cells can mediate the formation of ApoBDs via an apoptopodia-dependent or beaded apoptopodia-dependent process, respectively^13,15^ (Fig. 3a). Therefore, we next monitored the distribution of intracellular contents like DNA and mitochondria into ApoBDs generated by different cell types. ApoBDs generated from Jurkat T cells are more likely to contain a substantial amount of DNA compared to ApoBDs generated from THP-1 monocytes (Fig. 3d). Inversely, ApoBDs generated from THP-1 monocytes are more likely to contain a substantial amount of mitochondria than ApoBDs generated from Jurkat T cells (Fig. 3d). Collectively, these data suggest that ApoBDs are not homogeneous particles, and the levels and types of intracellular contents in ApoBDs could vary between different cell types, possibly due to differences in their mechanism of apoptotic cell disassembly.

### DNA and RNA are not packaged into separate ApoBDs

**Figure 4:**
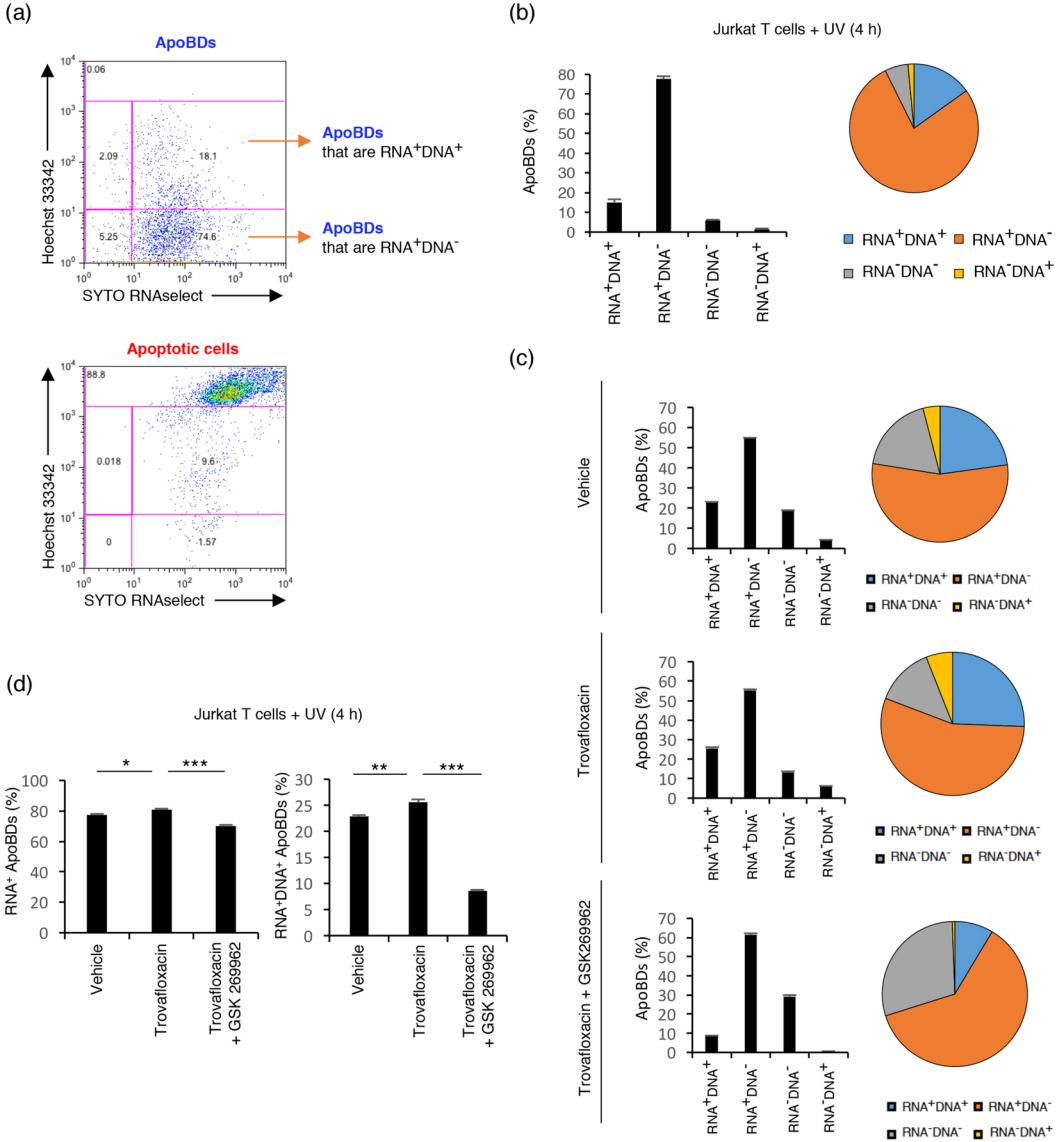
DNA and RNA are not separated into different ApoBDs. (**a**) Flow cytometry analysis showing the electronic gating strategy used to monitor the distribution of DNA and RNA into ApoBDs derived from apoptotic Jurkat T cells. Cells were stained with a combination of SYTO RNAselect, Hoechst 33342 and A5-APC. The gating of RNA^−^ DNA^−^ ApoBDs is determined based on unstained sample. (**b**) Left, bar chart and right, pie chart showing the percentage of RNA^+^DNA^+^, RNA^+^DNA^−^, RNA^-^DNA^+^, and RNA^−^DNA^−^ ApoBDs derived from UV treated Jurkat T cells (n = 3). (**c**) Jurkat T cells were treated with trovafloxacin (20 μM) alone or in combination with GSK 269962 (1 μM) to modulate the mechanism of ApoBD formation. The distribution of RNA and DNA into ApoBDs monitored as per (a) (n = 3). (**d**) The percentage of RNA^+^ and RNA^+^DNA^+^ ApoBDs are compared between different treatment groups as per (c) (n = 3). (**a–d**) Jurkat T cells were induced to undergo apoptosis by UV treatment. RNA^+^ and DNA^+^ ApoBDs represent ApoBDs containing a substantial amount of RNA and DNA, respectively. RNA^−^ and DNA^−^ ApoBDs represent ApoBDs containing no or very low amount of RNA and DNA, respectively. Data are representative of at least three independent experiments. *P < 0.05, **P < 0.01, ***P < 0.001, unpaired Student’s two-tailed t-test.

Previous studies suggested that DNA and RNA are packaged into separate ApoBDs from human HL-60 promyelocytic and MCF-7 breast adenocarcinoma cell lines, with over 90% of ApoBDs containing RNA having no detectable DNA, and vice versa as determined by confocal microscopy^22^. To examine whether DNA and RNA are indeed selectively packaged into different ApoBDs by flow cytometry, we first established a method to monitor the distribution of RNA into ApoBDs. Jurkat T cells were stained with SYTO RNAselect to monitor the localization of RNA prior to apoptosis induction^23^. ApoBDs containing a substantial amount of RNA, or ApoBDs containing no or very low amounts of RNA, were identified by the electronic gating strategy as shown in Supplementary Fig. 4. Localization of RNA into membrane blebs and ApoBDs according to SYTO RNAselect staining was also validated by confocal microscopy (Supplementary Figs 1d and 2c). To determine the co-distribution of RNA and DNA into ApoBDs, Jurkat T cells were stained with a combination of Hoechst 33342 and SYTO RNAselect prior to apoptosis induction, and stained with A5-APC to identify ApoBDs in the sample (see electronic gating strategy in Fig. 4a). Utilizing this approach, 80~90% of Jurkat T cell-derived ApoBDs were found to contain a substantial amount of RNA (Fig. 4b). Interestingly, around 20% of ApoBDs containing a substantial amount of RNA were found to harbor a substantial amount of DNA, whereas around 80% of ApoBDs containing a substantial amount of DNA had a substantial amount of RNA (Fig. 4b). It is worth noting that promoting ApoBDs formation through an apoptopodia-dependent mechanism (i.e. trovafloxacin treatment) increased the percentage of RNA^+^ ApoBDs and DNA^+^ RNA^+^ ApoBDs, whereas promoting ApoBDs formation through a beaded-apoptopodia-dependent mechanism (i.e. trovafloxacin and GSK 269962 treatment) reduced the percentage of RNA^+^ ApoBDs and DNA^+^ RNA^+^ ApoBDs (Fig. 4c and d). The co-distribution of DNA and RNA into ApoBDs was also examined using HL-60 cells and separate packaging of DNA and RNA into ApoBDs was not observed (Supplementary Fig. 5).

### CD146, CD45, CD3 and CD11b are optimal surface markers to distinguish HUVEC, THP-1 monocytes and Jurkat T cells by flow cytometry

**Figure 5:**
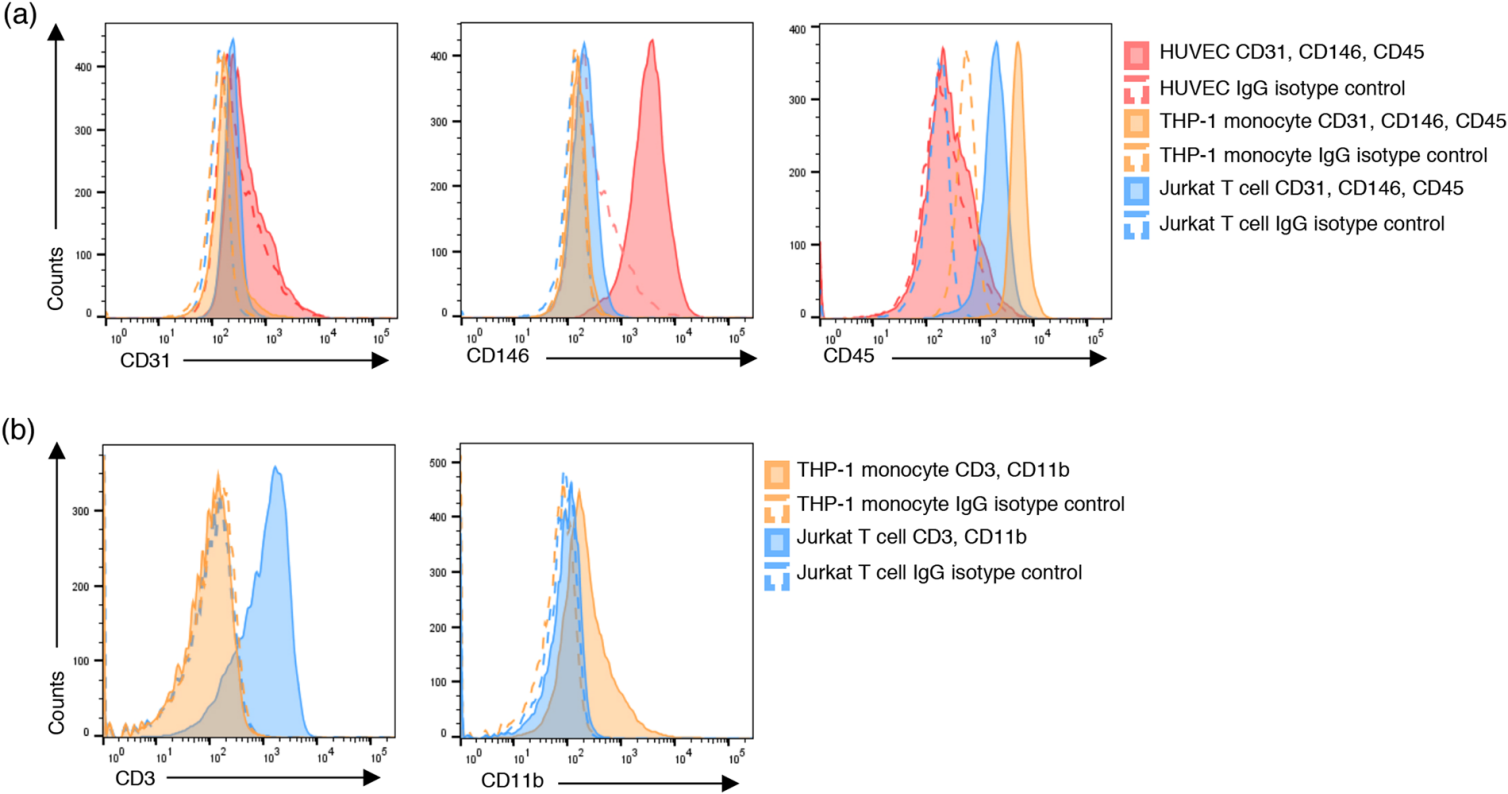
Establishing optimal surface cell markers on endothelial cells, monocytes and T cells. Representative histograms show surface expression of (**a**) CD31, CD146, CD45 on viable HUVEC, THP-1 monocytes and Jurkat T cells and (**b**) CD3 and CD11b expression on viable THP-1 monocytes and Jurkat T cells. Data are representative of at least three independent experiments.

In addition to ApoBD content, we were interested in the presence of cell type-specific markers on the surface of ApoBDs. In order to investigate whether ApoBDs derived from specific cell types carry cell-specific markers, we used HUVEC (human umbilical vein endothelial cells), THP-1 monocyte and Jurkat T cells as model cell lines. First, we determined the most suitable surface markers for the identification of these cells by flow cytometry. Platelet-endothelial cell adhesion molecule-1 (PECAM-1) or CD31 is widely used as an endothelial cell marker for flow cytometry analysis^24^. However, expression of CD31 by HUVEC used in the current study was low and therefore could not be used to distinguish endothelial cells from leukocytes (Fig. 5a, Supplementary Fig. 6). Unlike CD31, HUVEC demonstrated high expression of CD146 compared to THP-1 monocytes and Jurkat T cells, thus allowing for clear distinction of endothelial cells from leukocytes (Fig. 5a, Supplementary Fig. 6). To further distinguish endothelial cells from leukocytes, expression of the common leukocyte marker CD45 was high on both THP-1 monocytes and Jurkat T cells compared to HUVEC (Fig. 5a, Supplementary Fig. 6). Finally, differentiation of THP-1 monocytes from Jurkat T cells was based on CD11b and CD3 expression, cell surface markers for monocytes and T cells, respectively (Fig. 5b, Supplementary Fig. 6). Establishing these optimal surface marker combinations for HUVEC, THP-1 monocytes and Jurkat T cells will enable the characterization of these surface markers on ApoBDs.

### Changes in cell type-specific surface marker expression during apoptosis and cell disassembly

**Figure 6:**
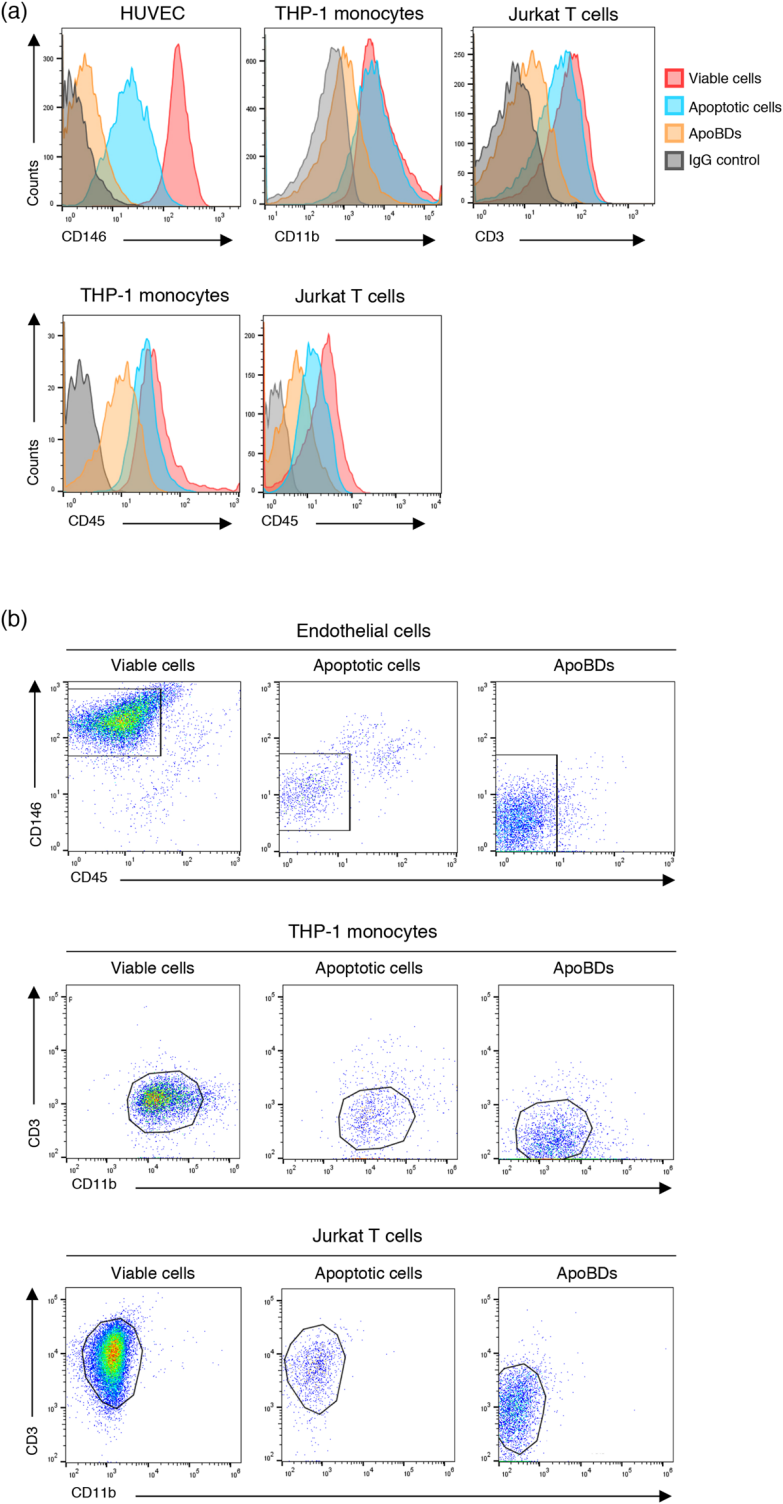
Changes in the expression of cell surface markers during apoptosis and cell disassembly. (**a**) Representative histograms showing the expression of cell surface markers CD146, CD11b, CD3 and CD45 on viable cells, apoptotic cells and ApoBDs from HUVEC, THP-1 monocytes and Jurkat T cells. (**b**) Electronic gating strategy for the identification of viable cells, apoptotic cells and ApoBDs derived from HUVEC, THP-1 monocytes and Jurkat T cells. Data are representative of at least three independent experiments.

A variety of modifications occur at the plasma membrane during apoptosis, in particular the exposure of phosphatidylserine at the outer leaflet of the plasma membrane to enable recognition by phagocytes^25^. However, whether the expression of cell type-specific surface markers changes during the progression of apoptosis and disassembly is not well defined. Once optimal surface markers including CD146, CD45, CD3 and CD11b were established (as described above), their expression on viable cells, apoptotic cells and ApoBDs (identified according to Supplementary Fig. 7) from the respective cell types was investigated. CD146, CD11b, CD45 and CD3 on HUVEC, THP-1 monocytes and Jurkat T cells respectively, were highly expressed on viable cells, with lower expression on apoptotic cells (Fig. 6a, Supplementary Fig. 8). Importantly, although expression was low compared to apoptotic and viable cells, ApoBDs from each cell type showed expression of respective surface markers (Fig. 6a). It should be noted that the expression of these markers on the surface of ApoBDs was confirmed by confocal microscopy (Supplementary Fig. 9). Based on these characteristics, an electronic gating strategy was established for viable cells, apoptotic cells and ApoBDs from HUVEC, THP-1 monocytes and Jurkat T cells to enable identification of the cell origin of ApoBDs (Fig. 6b). These data demonstrate that ApoBDs express surface markers from the cell of origin. However, since the expression of cell type specific markers on ApoBDs is relatively low, caution must be taken when establishing gating strategies for flow cytometric analysis.

### Identification of cell type specific ApoBDs in a mixed culture

**Figure 7:**
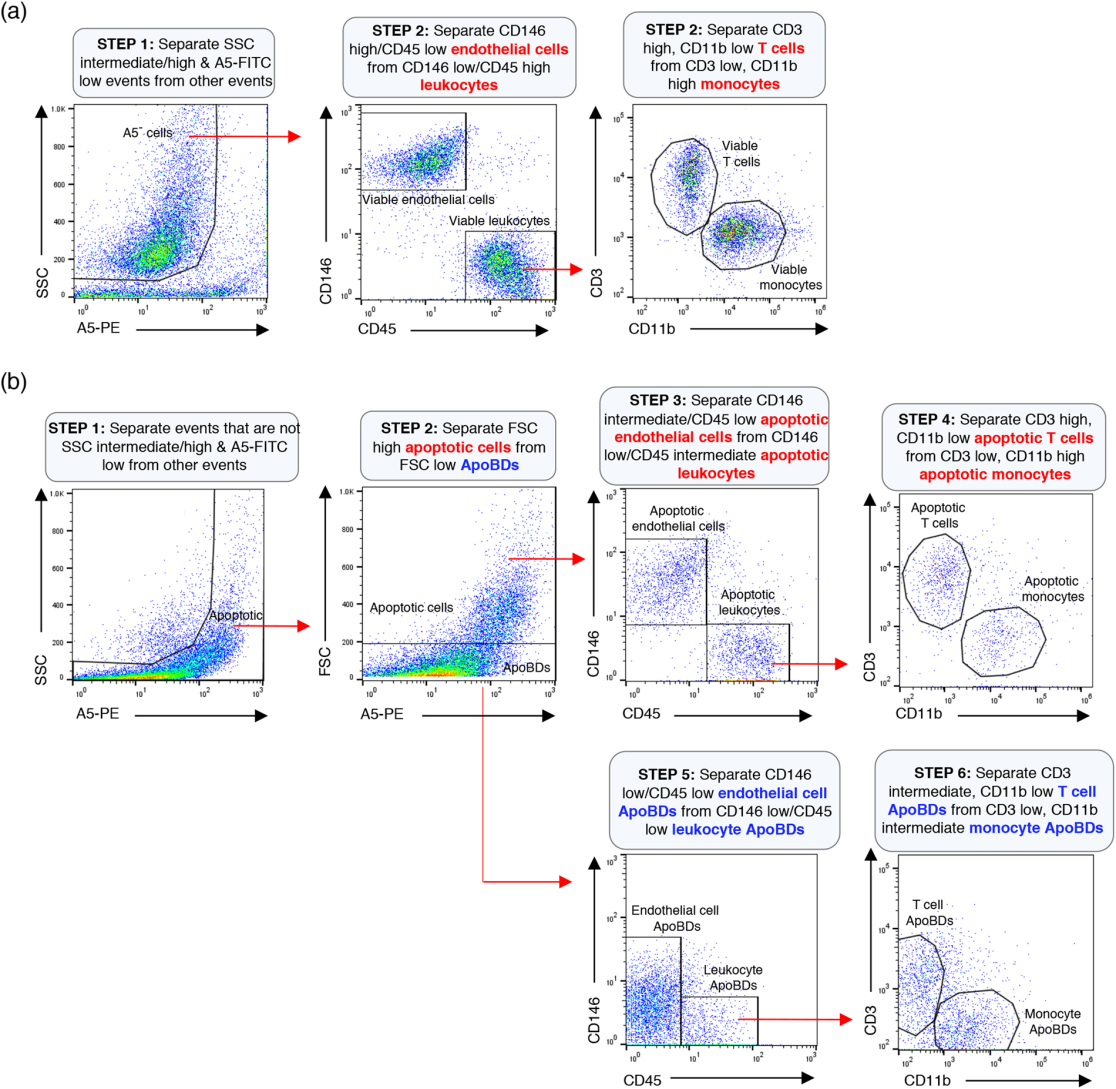
Identification of cell type specific ApoBDs in mixed culture by flow cytometry. (**a**) Electronic gating strategy used to identify viable endothelial cells, monocytes and T cells based on CD146, CD45, CD3 and CD11b surface expression. (**b**) Electronic gating strategy used to identify apoptotic cells and ApoBDs from endothelial cells, monocytes and T cells based on CD146, CD45, CD3 and CD11b surface expression. Data are representative of at least three independent experiments.

Having established a strategy to identify ApoBDs generated from endothelial cells, monocytes and T cells by flow cytometry, we were interested to know if respective surface markers could be used to distinguish cell type-specific ApoBDs in co-culture containing all three cell types. Using a mixed culture containing viable HUVEC, THP-1 monocytes and Jurkat T cells together with the gating strategy as established above, CD146^high^/CD45^low^ viable endothelial cells were separated from CD146^low^/CD45^high^ viable leukocytes (Fig. 7a). CD45^high^ viable leukocytes were further separated into CD3^high^/CD11b^low^ viable T cells and CD3^low^/CD11b^high^ viable monocytes (Fig. 7a). Next, in a mixed culture of apoptotic HUVEC, THP-1 monocytes and Jurkat T cells, CD146^high^/CD45^low^ apoptotic endothelial cells were separated from CD146^low^/CD45^high^ apoptotic leukocytes (Fig. 7b). CD45^high^ apoptotic leukocytes were further separated into CD3^high^/CD11b^low^ apoptotic T cells and CD3^low^/CD11b^high^ apoptotic monocytes (Fig. 7b). From the same apoptotic culture, CD146^intermediate^/CD45^low^ endothelial cell ApoBDs were separated from CD146^low^/CD45^intermediate^ leukocyte ApoBDs (Fig. 7b). Leukocyte ApoBDs were further separated into CD3^intermediate^/CD11b^low^ T cell ApoBDs and CD3^low^/CD11b^intermediate^ monocyte ApoBDs (Fig. 7b). Through the use of optimal surface markers for a particular cell type and novel analytical approach, cell type-specific ApoBDs can be distinguished in a co-culture of multiple cell types by flow cytometry.

## Discussion

It is becoming increasingly clear that the formation of ApoBDs during apoptosis is a highly regulated process^4,13,15^. However, the distribution of intracellular contents into ApoBDs is not well defined, possibly due to the lack of methodologies that could rapidly and accurately study this process. The data presented here demonstrate the ability to monitor and quantify the distribution of intracellular contents, including DNA, RNA and mitochondria, into ApoBDs efficiently by flow cytometry. It should be noted that other intracellular contents (e.g. other organelles or specific molecules) in ApoBDs could also be monitored using a similar approach if suitable staining or tracking methods are available. Likewise, similar approaches could be used for both adherent and non-adherent cells, as well as primary cells. Furthermore, utilizing the described flow cytometry-based approach, this work also establishes the concept that ApoBDs are not just one homogeneous identity, whereby subsets of ApoBDs can be defined based on their intracellular contents. This is particularly relevant for downstream functions of ApoBDs as different subsets of ApoBDs may exhibit different functions. For example, not all ApoBDs could mediate the transfer of autoantigens, cytokines or microRNAs to recipient cells. Furthermore, whether phagocytes could recognize and clear different subsets of ApoBDs differently remains to be determined.

As described in our recent studies^4,15,16^, apoptotic cell disassembly is regulated by three distinct morphological steps, namely membrane blebbing (Step 1), apoptotic protrusion formation (Step 2) and fragmentation to generate ApoBDs (Step 3). Importantly, apoptotic cells can utilize different mechanisms to generate ApoBDs depending on the activity of certain molecular factors^13,15,17^. In particular, manipulating the activity of key regulators of cell disassembly, such as ROCK1 and PANX1, can determine whether apoptotic cells will generate ApoBDs via apoptopodia (separate membrane blebs to form ApoBDs) or beaded apoptopodia (fragmentation of the apoptotic membrane protrusion to form ApoBDs)^13^. Using the flow cytometry method described in this study, we have provided evidence to support the idea that the content in ApoBDs could be influenced by the mechanism of their formation. For example, ApoBDs generated via beaded apoptopodia are less likely to contain a substantial amount of DNA compared with ApoBDs generated via apoptopodia. Thus, the distribution of intracellular contents into ApoBDs is not simply a stochastic process and could be regulated by the mechanism of apoptotic cell disassembly. Since different cell types can utilize different mechanisms to undergo cell disassembly during apoptosis^4,13–16^, ApoBDs generated by different cell types are likely to have different effects on physiological processes as well as the progression of certain diseases. It is interesting to note that previous studies have also proposed the packaging of intracellular contents such as DNA and RNA into ApoBDs is regulated, and DNA and RNA are segregated into different ApoBDs during apoptosis^22^. However, the data presented in this study suggest that although the distribution of intracellular contents into ApoBDs is a regulated process, DNA and RNA are not partitioned into separate ApoBDs. The discrepancy between previous work^22^ and our current study can be attributed to how the ApoBDs were handled prior to analysis (e.g. fixed versus not fixed, cytocentrifuged versus not cytocentrifuged), and how ApoBDs were analysed (e.g. microscopy analysis based on ‘body-like structures’ versus flow cytometry analysis based on A5 staining as well as relative size and complexity/granularity).

Until now, the identification of ApoBDs in cell culture samples has largely been based on characteristics like phosphatidylserine exposure, as well as particle size and granularity^19^. In order to further study and characterize the function of ApoBDs in various disease settings, it is necessary to be able to accurately identify these vesicles. Here, we demonstrate that in addition to intracellular contents, ApoBDs can also be identified and subclassed based on the cell of origin from which they arise. Through analysis of surface marker expression on HUVEC, THP-1 monocytes and Jurkat T cells, it was observed that the expression of cell type specific markers (e.g. CD146, CD45 and CD3) are reduced on apoptotic cells and ApoBDs compared to viable cells. Although why these cell type specific markers are reduced on the surface of apoptotic cells is unclear, the expression of these markers was sufficient to identify different subsets of ApoBDs in mixed culture based on their cell origin. Notably, it is possible to identify the origin of ApoBDs from body fluids by flow cytometry if appropriate surface markers and gating strategies are established.

Collectively, the flow cytometry-based approaches described in this study could be used to monitor the formation of different subsets of ApoBDs, and better understand their function under physiological and pathological conditions.

## Electronic supplementary material

Supplementary Figures
