# Supplementary material for: Determining the contents and cell origins of apoptotic bodies by flow cytometry
Source: Sci Rep. 2017 Oct 31;7:14444. doi: 10.1038/s41598-017-14305-z (PMC5663759; doi:10.1038/s41598-017-14305-z)
Supplement: Supplementary file 1 — Supplementary Figures [file 41598_2017_14305_MOESM1_ESM.pdf]

Determining the contents and cell origins of apoptotic bodies by flow cytometry

Lanzhou Jiang*, Stephanie Paone*, Sarah Caruso, Georgia K Atkin-Smith, Thanh Kha Phan, Mark D Hulett & Ivan K H Poon

Supplementary data

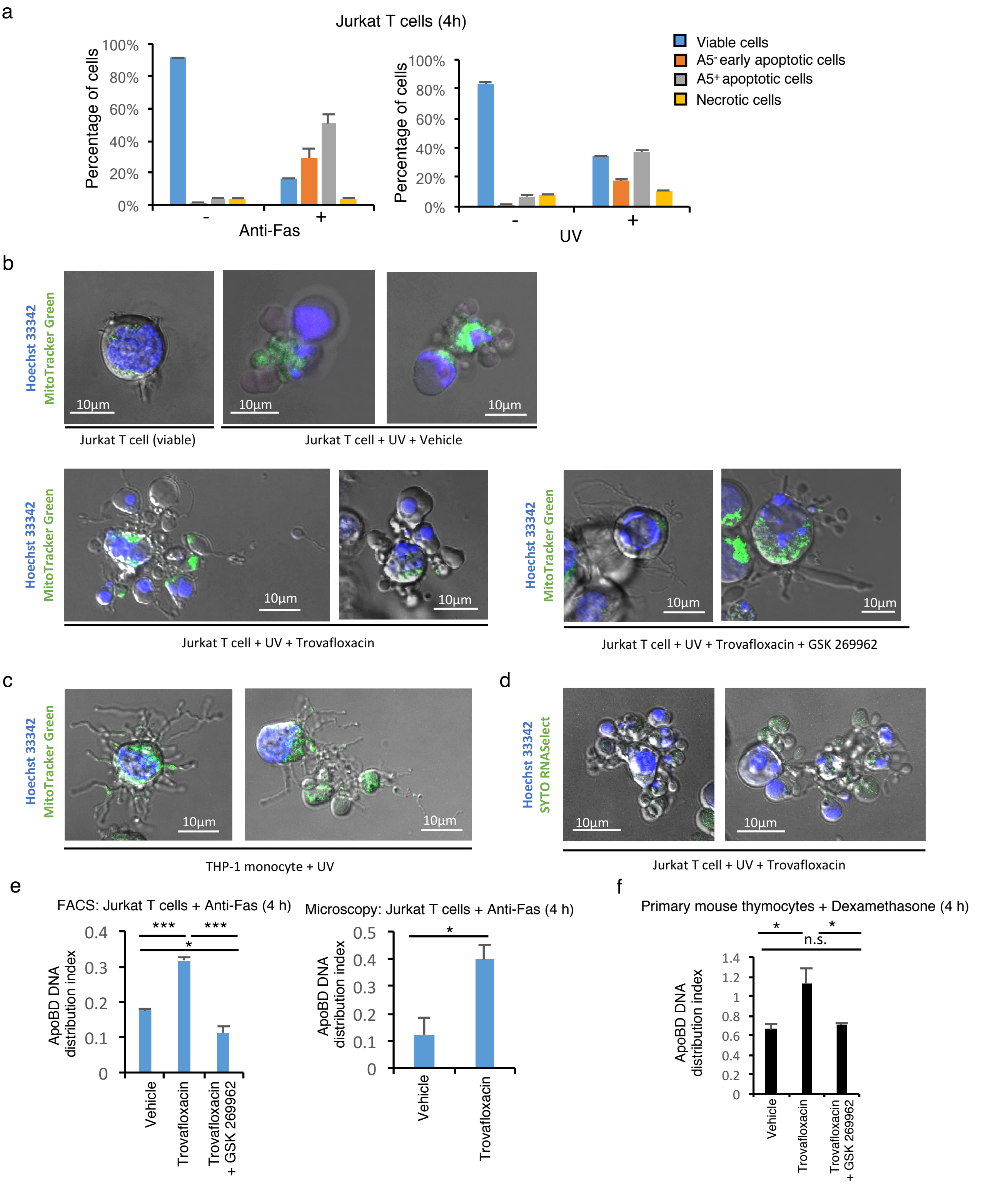

Supplementary Figure 1. Distribution of intracellular contents into ApoBDs is associated with the mechanism of apoptotic cell disassembly. (**a**) Apoptosis was induced by UV or anti-Fas treatment and Jurkat T cells were incubated for 4 h. The levels of viable cells, $A5^-$ apoptotic cells, $A5^+$ apoptotic cells and necrotic cells monitored by flow cytometry (n=3). Live confocal microscopy images showing the localization of Hoechst 33342 and MitoTracker Green staining in (**b**) Jurkat T cells undergoing apoptotic cell disassembly without drug treatment (top), or treated with trovafloxacin (PANX1 inhibitor, 20 µM) (bottom left), or treated with both trovafloxacin (20 µM) and GSK 269962 (ROCK1 inhibitor, 1 µM), and (**c**) THP-1 monocytes undergoing apoptotic cell disassembly. (**d**) Live confocal microscopy images showing the localization of Hoechst 33342 and SYTO RNAselect staining in Jurkat T cells undergoing apoptotic cell disassembly treated with trovafloxacin (20 µM). (**b-d**) Jurkat T cells and THP-1 monocytes cells were induced to undergo apoptosis by UV irradiation. (**e**) Jurkat T cells were treated with trovafloxacin (20 µM) alone or in combination with GSK 269962 (1 µM) to modulate the mechanism of ApoBD formation. The distribution of DNA in ApoBDs was monitored by both flow cytometry (left panel) and live confocal microscopy (right panel; ApoBD DNA distribution index = DNA^+ ApoBDs / DNA^- ApoBDs). Jurkat T cells were induced to undergo apoptosis by anti-Fas (n=3). **(f)** Primary mouse thymocytes are treated with trovafloxacin (20 µM) alone or in combination with GSK 269962 (1 µM) to modulate the mechanism of ApoBD formation. The distribution of DNA into ApoBDs quantified based on ApoBD DNA distribution index. Thymocytes were treated with dexamethasone (50 µM) *ex vivo* to induce apoptosis (n=3). $^*P < 0.05$, $^{**}P < 0.01$, $^{***}P < 0.001$, unpaired Student's two-tailed t-test. Data are representative of at least three independent experiments.

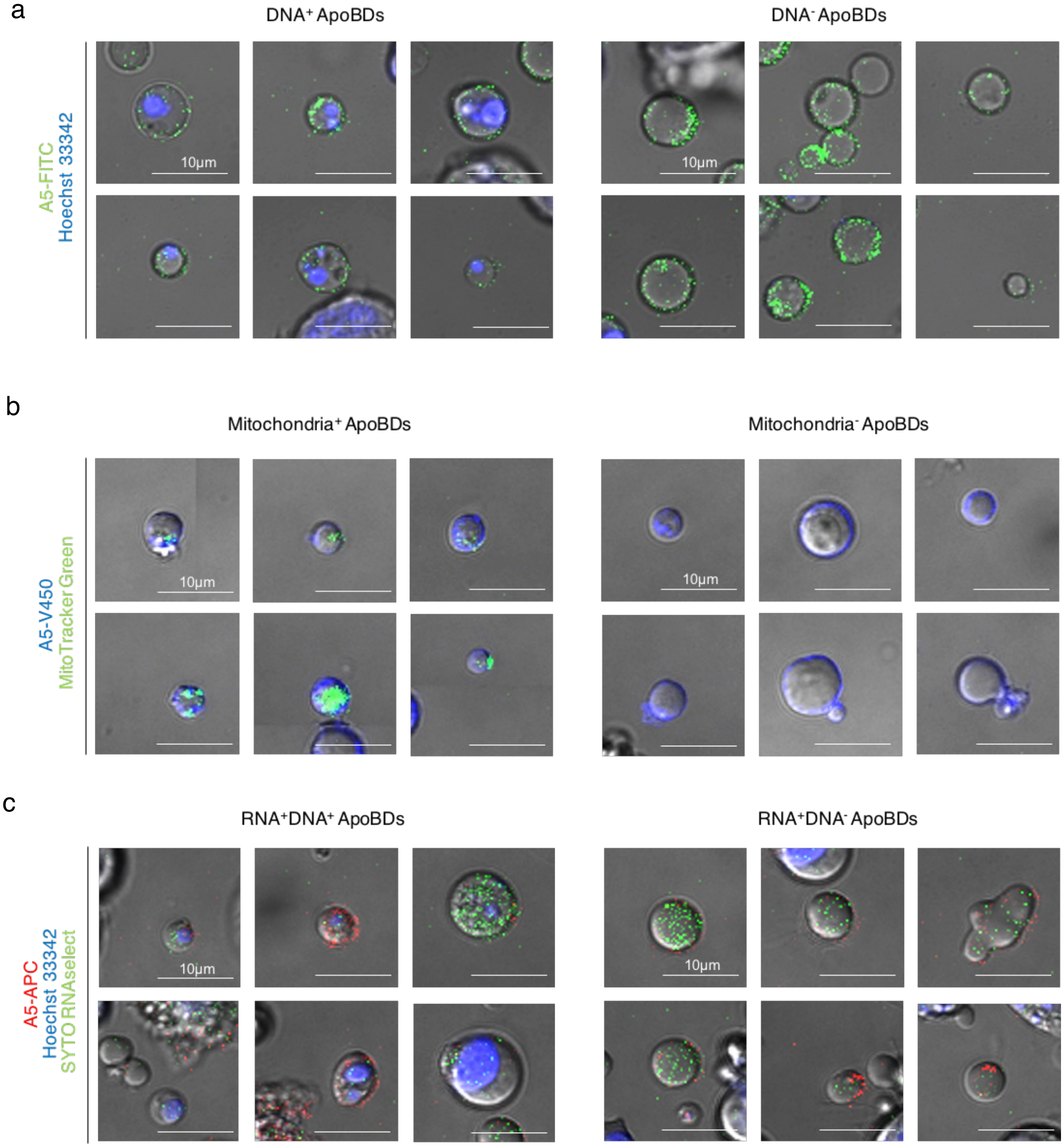

Supplementary Figure 2. Different subsets of ApoBDs can be identified based on intracellular contents by flow cytometry. (**a**) Live confocal microscopy images of ApoBDs from Jurkat T cells stained with Hoechst 33342 and A5-FITC. (**b**) Live confocal microscopy images of ApoBDs from Jurkat T cells stained with MitoTracker Green and A5-V450. (**c**) Live confocal microscopy images of ApoBDs from Jurkat T cells stained with Hoechst 33342, SYTO RNAselect and A5-APC. (**a-c**) Jurkat T cells were induced to undergo apoptosis by UV irradiation and treated with Trovafloxacin (20 µM) to promote apoptopodia-dependent apoptotic cell disassembly. Data are representative of at least three independent experiments.

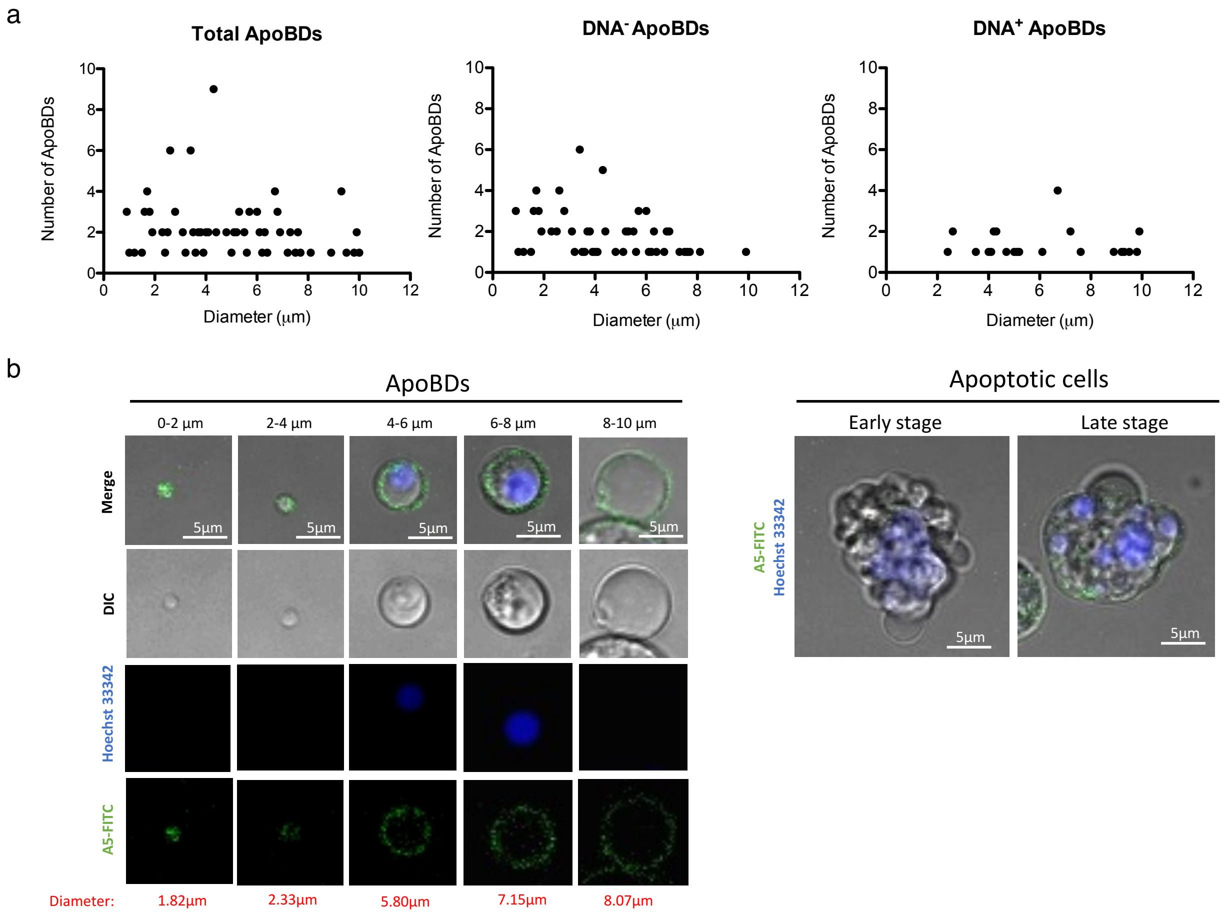

Supplementary Figure 3. Monitoring the size distribution of ApoBDs by confocal microscopy. (**a**) Diameter (µm) of ApoBDs generated from Jurkat T cells after 4 h of anti-Fas treatment in the presence of trovafloxacin (20 µM). (**b**) Live confocal microscope images showing ApoBDs of different sizes and apoptotic cells. A5-FITC and Hoechst 33342 staining are shown in green and blue, respectively. Data are representative of at least three independent experiments.

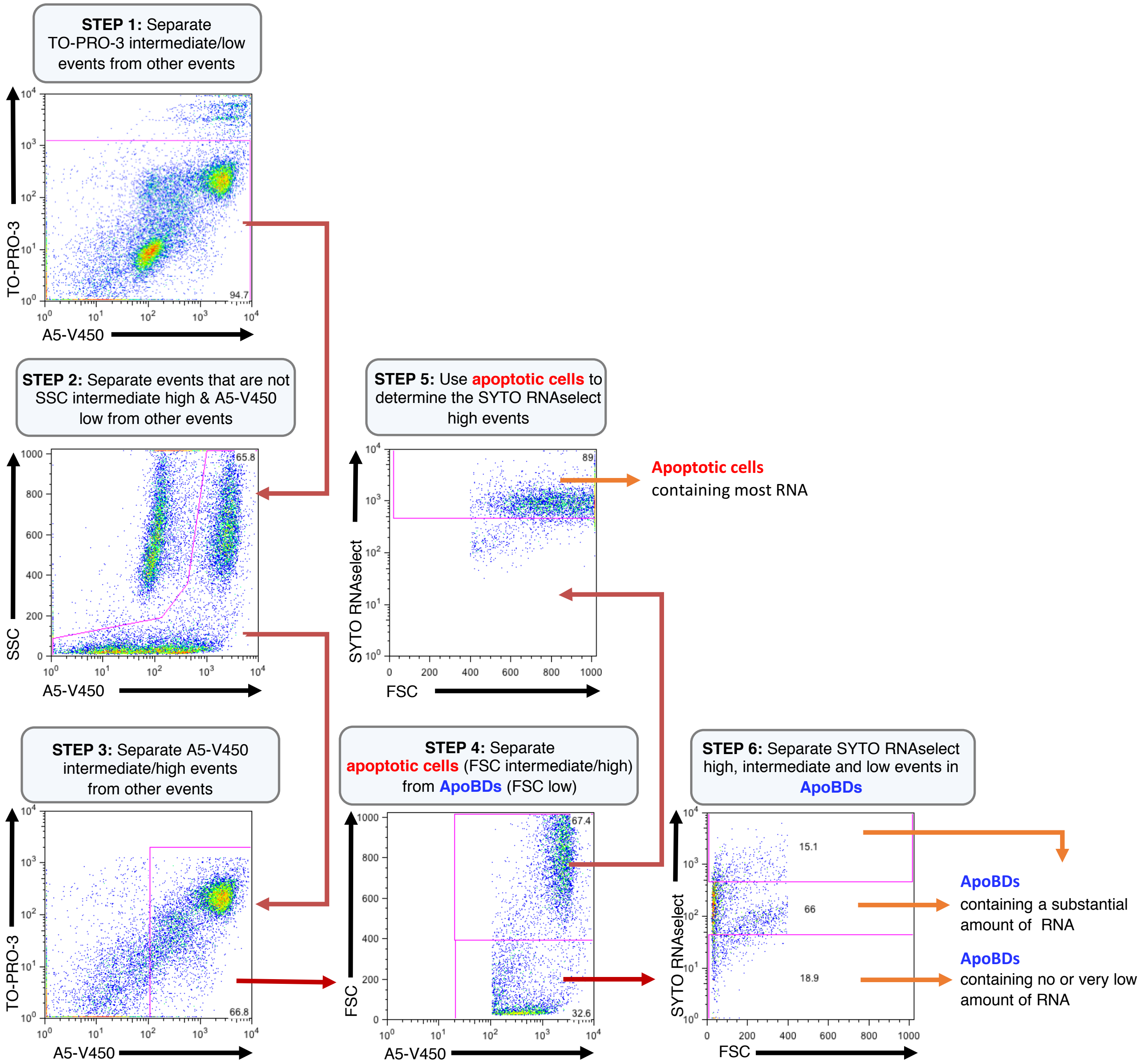

Supplementary Figure 4. Electronic gating strategy for analysis of RNA distribution in ApoBDs generated from Jurkat T cells. Flow cytometry analysis showing the six-stage electronic gating strategy used to identify ApoBDs with different amount of RNA from a sample containing a mixture of viable cells, apoptotic cells, necrotic cells, debris and ApoBDs. Data are representative of at least three independent experiments.

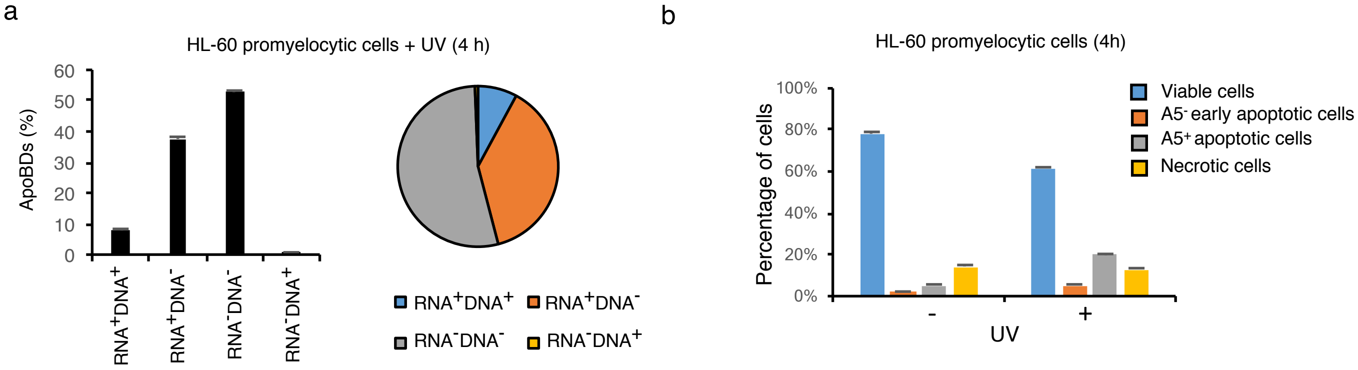

Supplementary Figure 5. DNA and RNA are not separated into different ApoBDs generated from HL-60 cells. (**a**) HL-60 cells were induced to undergo apoptosis by UV treatment. RNA^+ and DNA^+ ApoBDs represent ApoBDs containing a substantial amount of RNA and DNA, respectively. RNA^- and DNA^- ApoBDs represent ApoBDs containing no or very low amount of RNA and DNA, respectively. (**b**) HL-60 cells were induced to undergo apoptosis by UV irradiation (4 h) and the levels of viable cells, $A5^-$ apoptotic cells, $A5^+$ apoptotic cells and necrotic cells monitored by flow cytometry (n=3). Data are representative of at least three independent experiments.

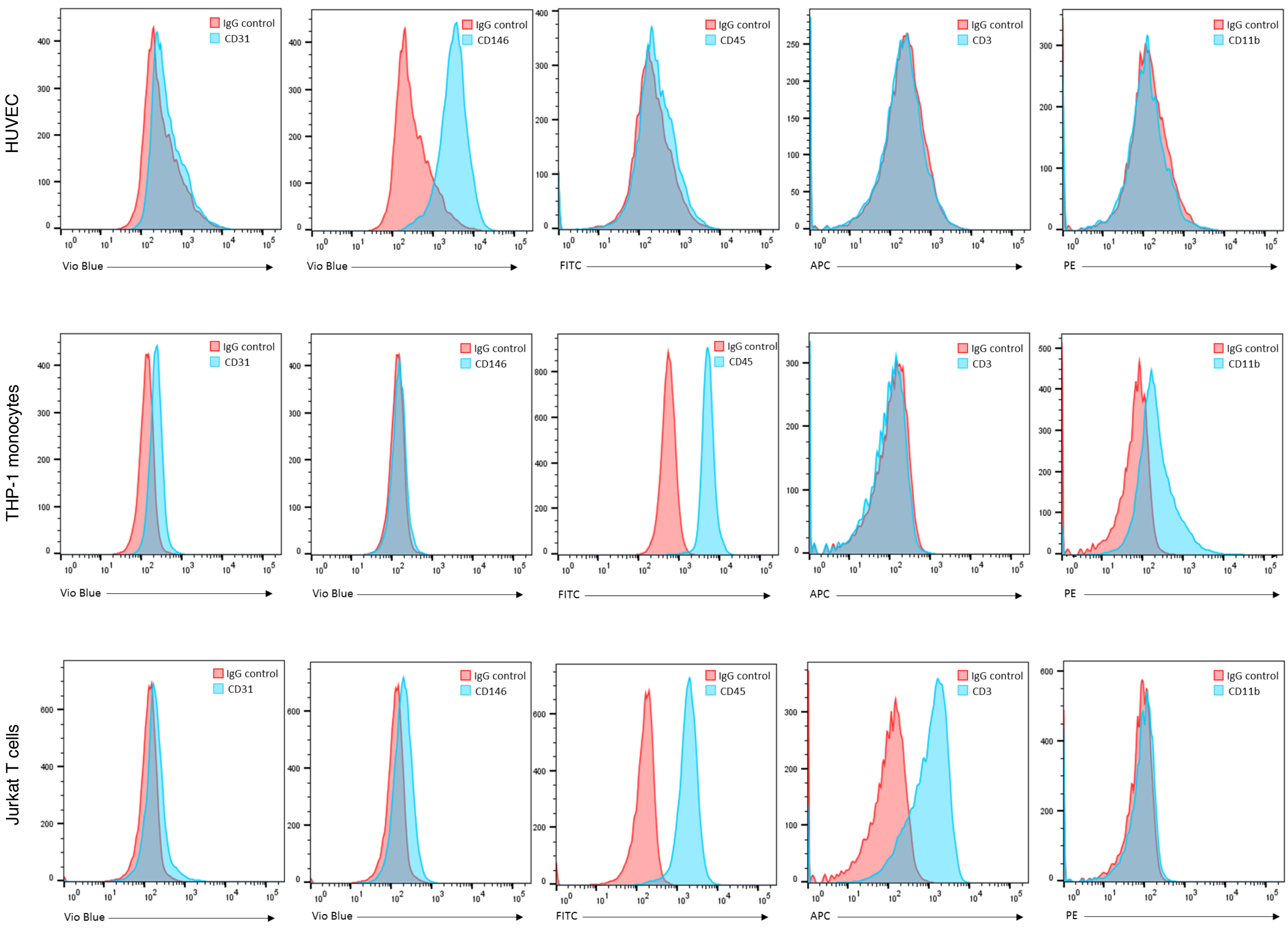

Supplementary Figure 6. Isotype controls for all antibodies used to monitor cell surface markers on HUVEC, THP-1 monocytes and Jurkat T cells. Representative histograms show surface expression of CD31, CD146, CD45, CD3 and CD11b on viable HUVEC, THP-1 monocytes and Jurkat T cells. Data are representative of at least three independent experiments.

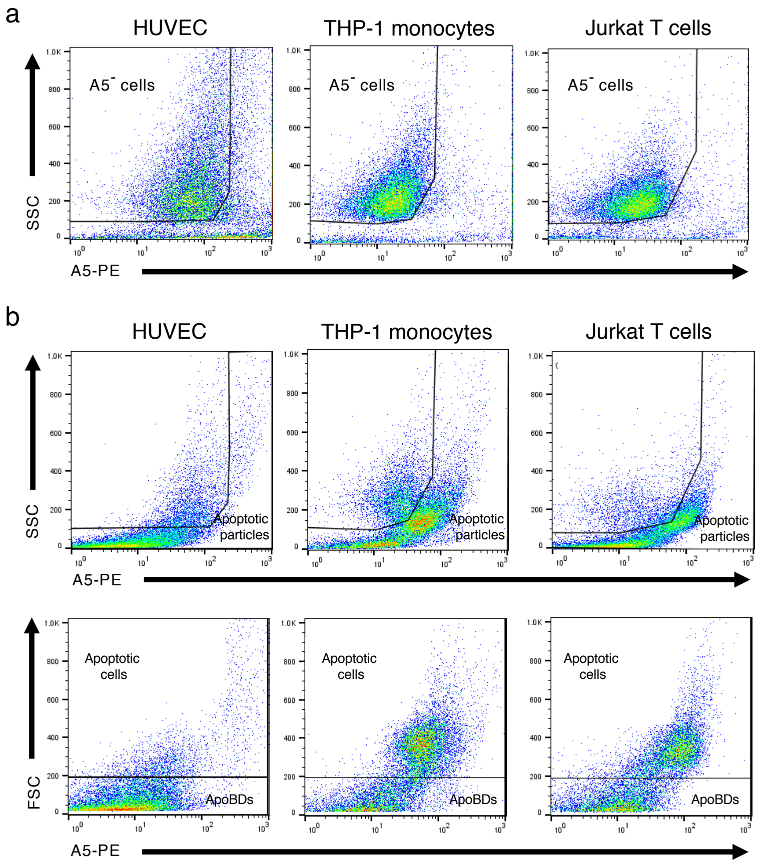

Supplementary Figure 7. Electronic gating strategy for analysis of cell viability and ApoBD formation. Electronic gating strategy used to define viable cells, apoptotic cells and ApoBDs in (**a**) viable and (**b**) apoptotic HUVEC, THP-1 and Jurkat T cell samples. HUVEC, THP-1 monocytes and Jurkat T cells were induced to undergo apoptosis by UV irradiation. Data are representative of at least three independent experiments.

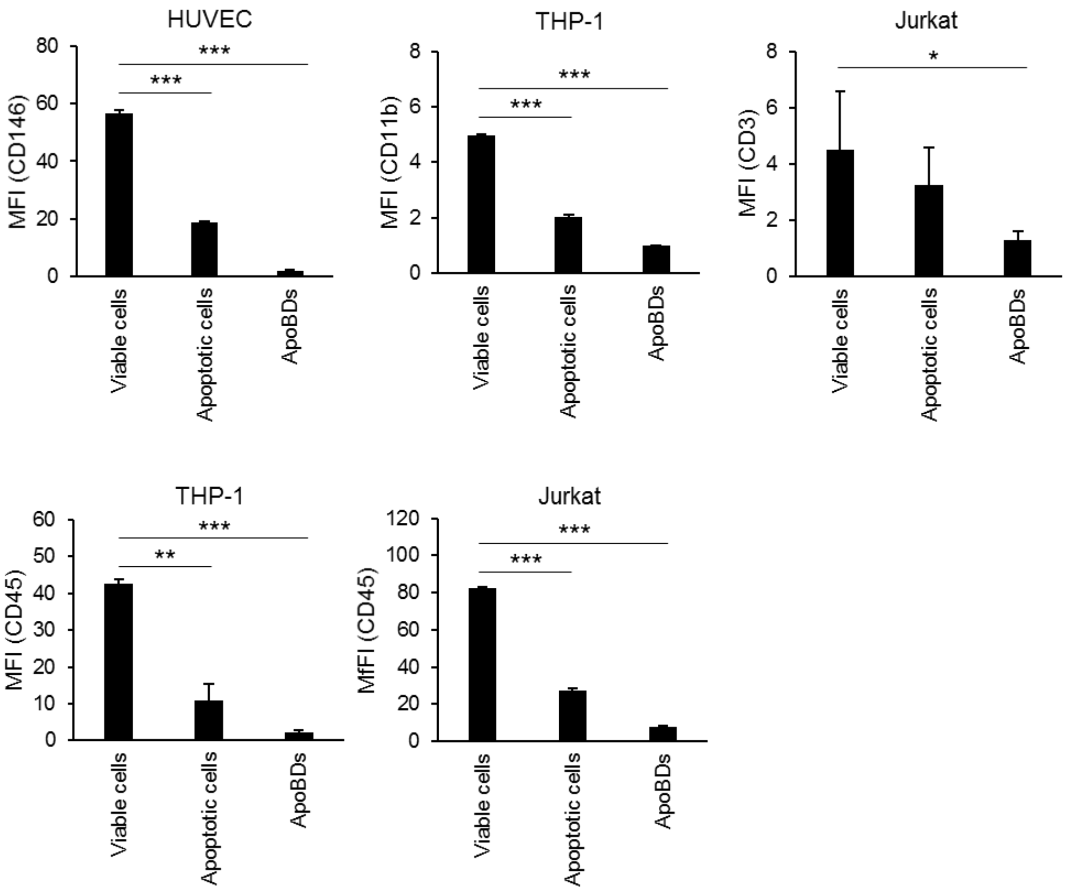

Supplementary Figure 8. Statistical analysis of surface marker changes during apoptosis and cell disassembly. Fold change in median fluorescence intensity (MFI) of CD146, CD11b, CD3 and CD45 on HUVEC, THP-1 monocytes and Jurkat T cells in viable cells, apoptotic cells and ApoBDs. Data are representative of at least three independent experiments. * *P*<0.05, ***P*<0.01, ****P*<0.001, unpaired Student's two-tailed t-test.

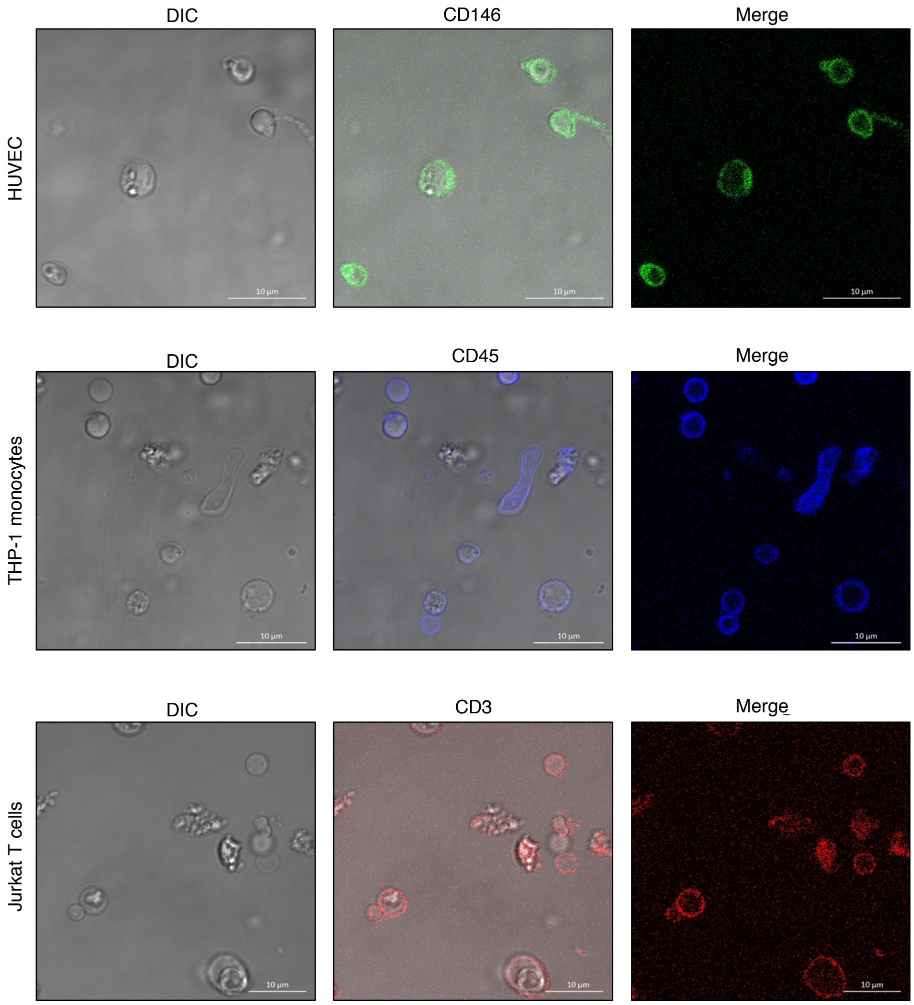

Supplementary Figure 9. Monitoring surface markers on ApoBDs by confocal microscopy. Live confocal microscopy images of ApoBDs from HUVEC, THP-1 monocytes and Jurkat T cells stained with anti-CD146, anti-CD45 and anti-CD3 respectively. Data are representative of at least three independent experiments.
